# Severe fever with thrombocytopenia syndrome virus was found in Northern Jiangxi Province, China

**DOI:** 10.3389/fmicb.2024.1500146

**Published:** 2024-12-04

**Authors:** Haijun Hu, Ying He, Fei Chen, Zhanbin Liu, Wei Wang, Shu Yang, Ke Qian, Zhuan Zhan, Yangping Guo, Hui Li, Weiqing Zheng

**Affiliations:** ^1^The Collaboration Unit for State Key Laboratory of Infectious Disease Prevention and Control, Jiangxi Provincial Health Commission Key Laboratory of Pathogenic Diagnosis and Genomics of Emerging Infectious Diseases, Nanchang Center for Disease Control and Prevention, Nanchang, Jiangxi, China; ^2^Department of Infectious Diseases, The First Affiliated Hospital of Nanchang University, Nanchang, Jiangxi, China; ^3^Nanchang Police Dog Base of the Ministry of Public Security, Nanchang, Jiangxi, China; ^4^Center for Disease Control and Prevention of Donghu District, Nanchang, Jiangxi, China; ^5^NHC Key Laboratory of Tropical Disease Control, School of Tropical Medicine, Hainan Medical University, Haikou, Hainan, China

**Keywords:** severe fever with thrombocytopenia syndrome, genotype, C4 and C5, genetic recombination, Jiangxi

## Abstract

**Introduction:**

Severe fever with thrombocytopenia syndrome (SFTS) is an emerging infectious disease discovered in China in 2009. SFTS monitoring has been carried out since 2010 in mainland China. In recent years, human infection with SFTS virus (SFTSV) has frequently been detected in Jiujiang of Jiangxi Province, Central China.

**Methods:**

Sera of SFTS surveillance cases and samples collected from humans, animals and ticks surrounding the cases were used to detect SFTSV RNA by real-time RT-PCR. SFTSV-positive samples were further subjected to sequencing and analysis of the S, M, and L segments of SFTSV.

**Results:**

Four patients were positive for SFTSV infection. However, the subjects like humans, animals and ticks around the cases were all detected as negative for the virus infection. Phylogenetic analysis of the partial segments revealed that SFTSVs in three patients from Jiujiang were clustered with genotype C4 and C5 on the L and M segment-established phylogenetic tree; however, phylogenetic analysis of the S segment showed that the strains were grouped into genotype J1. These results suggested that the S, M, and L segments of these strains underwent segmental reassortment, which was later supported by recombination signal detection. The reassortment event was detected by at least four methods with a significance level of *p* value <0.05. In addition, the RDP recombination consensus score (RDPRCS) was greater than 0.40. To avoid poor tree topology support with the partial S, M, and L segments, we further performed analysis of the complete genome of SFTSV. The full-length L, M, and S segment sequences of the strains were consistently clustered into two genotypes, namely the genotype C5 and the genotype C4. The strains belonging to genotype C5 were detected for recombination signal with moderate confidence by all the six methods, with a significance level of p value <0.05 and an RDPRCS of 0.41. The recombination event might have occurred between the minor parent (2011YPQ11, C3/C3/C3) and major parent (SPL053A, J1/ J1/ J1). However, there was no genetic recombination detected in the strains belonging to genotype C4. The event was detected by only two methods with a significance level of *p* value <0.05.

**Conclusion:**

Two genotypes of SFTSVs were identified in Jiujiang City, Jiangxi Province of Central China, and they were genotype C5 and genotype C4. The genotype C5 underwent genetic recombination in this region, with the minor parent of the strain 2011YPQ11 and the major parent of the strain SPL053A.

## 1 Background

Severe fever with thrombocytopenia syndrome (SFTS) is an acute tick-borne infectious disease with a high case mortality risk, ranging from 6.18 to 27% (Casel et al., 2021). The disease is caused by the SFTS virus (SFTSV), a novel Bunyavirus. In China, SFTS has been endemic in many locations since the first case was reported in 2009 (Yu et al., 2011), and the majority of cases were clustered with four geographical areas: the Changbai Mountain area (Cluster I), the Jiaodong Peninsula (Cluster II), the Taishan Mountain area (Cluster III), and the Huaiyangshan Mountain area (Cluster IV) (Miao et al., 2021). So far, the annual number of cases has been continually increasing, which raises significant public health awareness in China (Li et al., 2022). In recent years, patients with SFTSV infection have also been increasingly found in the Mufu mountains of Jiujiang City, Jiangxi Province. However, infections of SFTSV in Jiangxi Province have not been well investigated until now; furthermore, characterization of molecular features of SFTSV in Jiangxi remains elusive. Here, we phylogenetically analyzed the genomes of SFTSV strains from Jiangxi in combination with other strains from China and Japan available in GenBank. The results suggested potential recombinants of SFTSV appearing in Jiangxi strains.

## 2 Materials and methods

### 2.1 Sample collection from patients and ticks, animals and individuals in the affected areas at a high risk

Patients who were clinically diagnosed with symptoms as described previously (Hu et al., 2018), were considered suspected SFTS cases and thus were recruited in this study. Ten milliliter of blood samples were collected at the acute phase of suspected SFTS cases by the local CDC or hospital.

**Figure 1:**
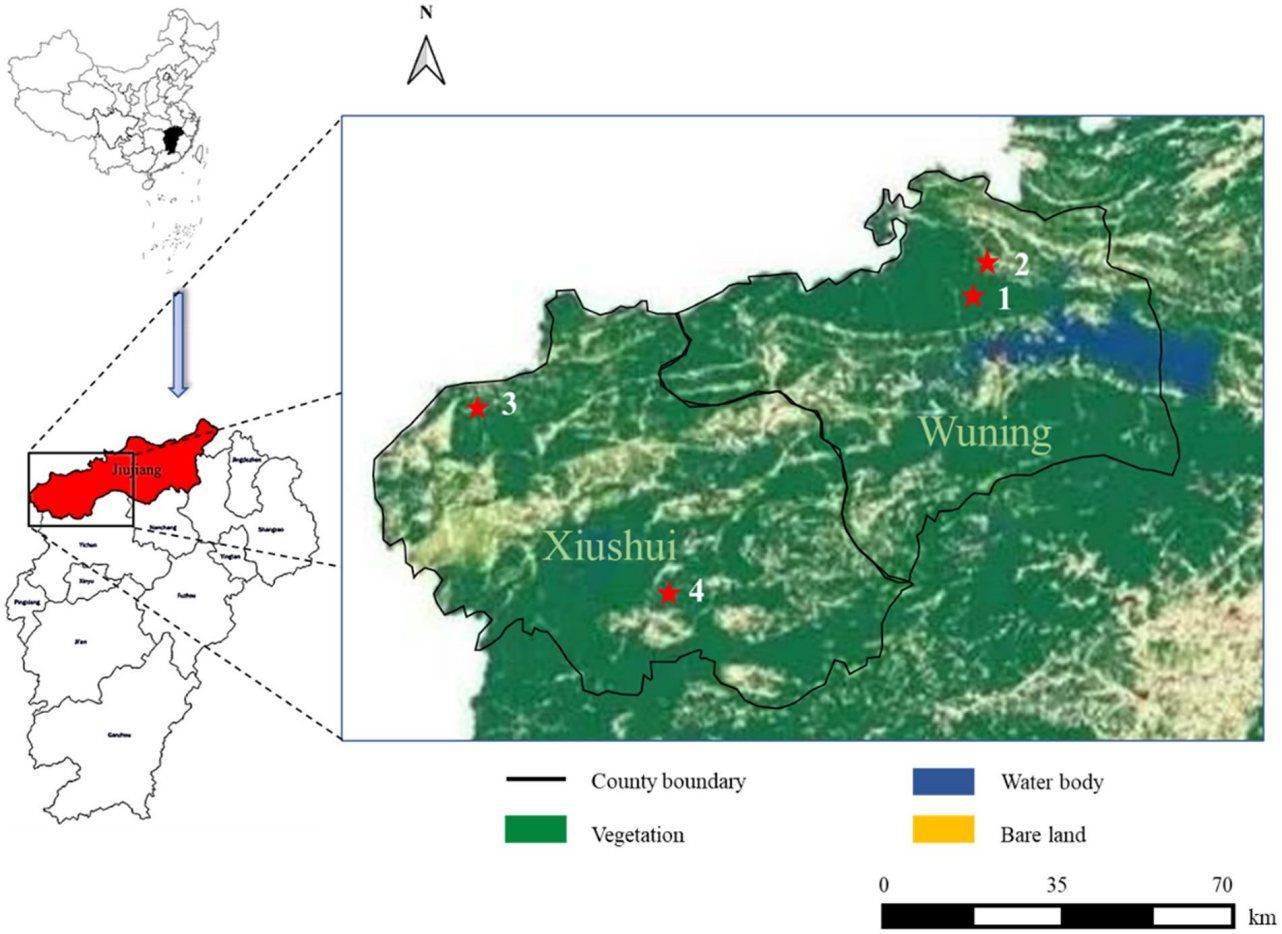
Severe fever with thrombocytopenia syndrome (SFTS) case distribution from this study in different land covers of Wuning and Xiushui County in Jiujiang City of Jiangxi Province from China. SFTS cases are shown with red filled pentagrams.

Ticks were directly plucked from infested animals or collected by flagging vegetation in the affected areas where patients were found (Figure 1). All collected ticks were maintained alive in cases at room temperature before the study. Tick species were identified morphologically (Chen and Yang, 2021) and confirmed by a molecular method (Lv et al., 2014).

Approximately 1–2 mL of blood samples of animals were collected from jugular veins depending on body size and serum was obtained after blood clotting, then stored in boxes with ice packs and transported to the laboratory along with the ticks where all were stored at −80°C until further processing. Serum samples of individuals at a high risk were collected from close contacts and the surrounding population when the patients were epidemiologically, molecularly, and clinically confirmed.

### 2.2 Total nucleic acid extraction

The tick pools of each sample location with the same species at the same stage were homogenized in a Tissuelyzer II (Qiagen, Germany) at 4°C and centrifuged at 10,000 × *g* at 4°C for 10 min. Fifty microliter aliquots of each supernatant were transferred and pooled together in fresh tubes. Twenty microliter of each human or animal serum were pooled. All supernatants and pooled sera were sterilized by filtration through 0.22-μm syringe filters, digested with 5 μL DNase at 20–30°C for 15 min, and immediately subjected to total nucleic acid automatic extraction in a QIAcube (Qiagen, Germany) using the RNeasy mini kit (Qiagen, Germany).

### 2.3 Quantitative real-time PCR (qRT-PCR)

Total RNA was extracted from the sera of patients, individuals at high risk, animals, and ticks using the QIAamp Viral RNA Mini Kit (Qiagen, Germany) according to the manufacturer’s instructions. The eluate (5 μL) was tested for SFTSV RNA using a commercial kit (Shuoshi Biotech., Jiangsu, China). The cutoff cycle-threshold (Ct) value for positive samples was set as 37 cycles.

### 2.4 Sequencing and analysis of the S, M, L segments of SFTSV

The SFTSV RNA was extracted from different samples with the QIAamp Viral RNA Mini Kit (Qiagen, Germany). The nucleic acids were reverse transcribed with the Reverse-Transcription Kit (TaKaRa, Dalian, China). To detect SFTSV RNA, we performed nested PCRs with a kit (Toyobo, Japan) and the amplified primers were listed in Table 1. Fragments covering the full-length L, M, and S segments of the SFTSV genome were amplified using walking primers designed based on the full-genome sequence of the detected SFTSV strain (Table 2). All PCR products were examined by electrophoresis on 1.5% agarose gels and the products with the targeted band were sent to a company for sequencing (Sangon, Shanghai, China).

**Table 1:** The specific primer sets used for partial SFTSV genome amplification.

Segment	Forward		Reverse		Size (bp)	Remark
	Name	Sequence	Name	Sequence		
L	L2136-F1	TGGATTGCATGGTGCGAATTG	L2135-R1	GATCAGATGACCTAGACTCAG	1020	Nested PCR
	Seq2-F2	GAACATTCCATGCCATCTCAG	Seq2-R2	GTGAGCTAAAAACCTTAGGTC	914	
M	MF3	GATGAGATGGTCCATGCTGATTCT	MF2	CTCATGGGGTGGAATGTCCTCAC	560	
S	Stest-F1	ATGTCAGAGTGGTCCAGGATT	Stest-R1	AAGGATTCCCTTGGCCTTCA	675	Nested PCR
	Stest-F2	TTGCAGTGGAGTTTGGTGAGC	Stest-R2	AAGGATTCCCTTGGCCTTCA	601	

**Table 2:** The specific primer sets used for whole SFTSV genome amplification.

Segment	Forward		Reverse		Size (bp)
	Name	Sequence	Name	Sequence	
L	LF-1F	ACACAGAGACGCCCAGATG	LF-1R	GAACCCCTCCTGACGAGACTAC	548
	LF-2F	CAACCACTAGGAGCCATAA	LF-2R	TGTCCCTCGATTCAATGATGT	605
	LF-3F	ATCACCAAGACCCTCAAAGC	LF-3R	TCCAACAAGATTGACACAGCTC	668
	LF-4F	GCAGCAAACCAGAAGAAAGA	LF-4R	CTCCATCTGGGTGTACCTTGT	706
	LF-5F	AGAGAGAAGTTGGGCGTGGC	LF-5R	AGGATGTTCTTGGTCCCAATCTC	660
	LF-6F	AGCTTCCTCAGAGCTGCTTG	LF-6R	CCTTAGGTCCACCATCATCTT	684
	LF-7F	GCCAAGAAGTGGAATCAGG	LF-7R	ATGAGGCCCCTCCTTCCAAGC	739
	LF-8F	TTCCACAGGCACTTGGTTAG	LF-8R	CGAAAGCAGTGGCGTGAGAG	707
	LF-9F	GCTGCCAACAAGAAGGAAC	LF-9R	TCTAGGCTAAAACCAGGGA	710
	LF-10F	TTAGGAACTTCATAGCCCACG	LF-10R	CCGTCAGTCCTTGATGCTGG	709
	LF-11F	GTAGTGATGCCAGGCTTTATG	LF-11R	CTTCTCCAAACTCTTCCACCTC	738
	LF-12F	GGTCAAAGCTCACAGAGATGG	LF-12R	AGACCGCCCAGATCTTAAGGAAATC	546
M	MF-1F	ACACAGAGACGGCCAACAATG	MF-1R	TCTCCTCAGGGATGGGTGTCA	586
	MF-2F	GATAGTTCCTGGGCCTTCATACAA	MF-2R	GAAACCRTAGCACTTTGGTCTGA	640
	MF-3F	GAGGCATCTGAGGCCAAGTG	MF-3R	CCTATTTCCTCATGGATCACTTGC	677
	MF-4F	TGGGGRTCATGGGTCATAGCTC	MF-4R	CTGCCCAATCATCAGAAAAGG	586
	MF-5F	GGAGTCCGGACTCAAAATGTC	MF-5R	GCGTCATCCACYCGTAGCTC	685
	MF-6F	TTAGCTCCCTGCAACCAGGC	MF-6R	CCACAAATTTTGGGACATCCAG	634
	MF-7F	GGGATGAGACTGCATTCAGTG	MF-7R	ACACAAAGACCGGCCAACACTTC	527
S	SF-1F	ACACAAAGACCCCCTTCATTTGG	SF-1R	GCTCATCATCCTCATCCAAGACAC	749
	SF-2F	CTCCTCCAGATAGAGTCACTTGCA	SF-2R	TGAAGCCACAACCAAGACCCT	646
	SF-3F	TAAACTTCTGTCTTGCTGGCTCC	SF-3R	ACACAAAGACCCCCAAAAAAGG	622

Sequences generated from this study and retrieved from GenBank were divided into the partial L, M, and S datasets, and the complete L, M, and S datasets for respective phylogenetic analysis. Comparative sequence analyses, including sequence alignments and best-fit substitution model selection, were performed using the ClustalW program of the Molecular Evolutionary Genetic Analysis (MEGA) software (version 7.0) (Tamura et al., 2013). Phylogenetic trees were constructed using the Maximum Likelihood method with a Kimura two-parameter model for analysis of the partial S, M, L, and the complete S segments of SFTSV. The Maximum Likelihood method with General Time Reversible model was employed for analysis of the complete M and L segments of SFTSV, which were provided in the MEGA software (version 7.0), and branch support was calculated based on 500 bootstrap replicates (Kimura, 1980; Saitou and Nei, 1987). Non-uniformity of evolutionary rates among sites were modeled using a discrete Gamma distribution (+G) with 5 rate categories.

### 2.5 Detecting recombination and reassortment events in SFTSV strains

Recombination and reassortment events were detected using the RDP software package, which integrates six recombination detection methods, including GENECONV, BootScan, MaxChi, Chimaera, SiScan, and 3Seq (Martin et al., 2015). To identify reassortment occurrences, the partial sequences of the L, M, and S segments from SFTSV strains in Supplementary Dataset 1 were sequentially concatenated and subsequently analyzed using the RDP software package. A reassortment event was characterized as a recombination breakpoint detected at the ends of segments (Nagarajan and Kingsford, 2011). For a recombination event to be confirmed, it had to fulfill two criteria outlined in the RDP manual: (i) the event must be validated by at least two methods with a significance level of *p*-value <0.05; and (ii) the RDP recombination consensus score (RDPRCS) must exceed 0.60. If an event met the first criterion but fell within the RDPRCS range of 0.4–0.6, it was considered a probable recombination; otherwise, it was disregarded. For recombination detection in the complete genome of SFTSV, the full-length L, M, and S segments from SFTSV strains stored in Supplementary Dataset 2 were sequentially concatenated and subsequently analyzed using the same methods as mentioned above.

### 2.6 GenBank data contribution

In the present study, 12 sequences were obtained and deposited in GenBank under accession numbers OR035637-OR035639, OR557464-OR557466, OR574960-OR574965, and OR915871-OR915873. We named the SFTSV strain from patient 1 SFTSV strain JX22-1 and successfully generated partial S segment (OR035639), M segment (OR035637), and L segment (OR035638). We termed the SFTSV strain from patient 2 SFTSV strain JX23WN and successfully generated partial S segment (OR557464), M segment (OR557465), and L segment (OR557466), and complete S segment (OR574963), M segment (OR574964), and L segment (OR574965). Patient 3 was infected by the SFTSV strain JX23XSH, and we successfully generated partial S segment (OR574960), M segment (OR574961), and L segment (OR574962), and complete S segment (OR915871), M segment (OR915872), and L segment (OR915873) from this strain.

## 3 Results

### 3.1 SFTSV detection in patients, humans living around the patients, and ticks and animals

A total of four patients were enrolled in this study, and their clinical symptoms were provided in Supplementary Table S1. From three of them, we successfully generated partial and/or complete segment sequences of SFTSV. From June 26 to 28, 2022, and from July 12 to 14, 2023, a total of 56 humans who live and work around the patients’ home environment were tested for SFTSV infection. Among these individuals, 43 reported tick bites and nine had multiple tick bites. The PCR results for SFTSV infection in all individuals tested were negative. A total of 91 ticks, 18 domestic animals (12 dogs, five goats, and one cat), and seven rodents found in the patients’ home environment were also sampled. Ticks were composed of three *Haemaphysalis longicornis*, seven *Haemaphysalis hystricis* and one *Ixodes granulatus* ticks. Eighty host-attached ticks were collected with tweezers and consisted of 45 *H. longicornis*, 32 *H. hystricis*, two *Haemaphysalis kitaokai* and one unidentified *Haemaphysalis* tick. Among all the collected ticks, only four were nymphs and the remaining were adults. SFTSV RNA was not detected in any of ticks and animals tested.

### 3.2 Genetic analysis of SFTSV in three patients

**Figure 2:**
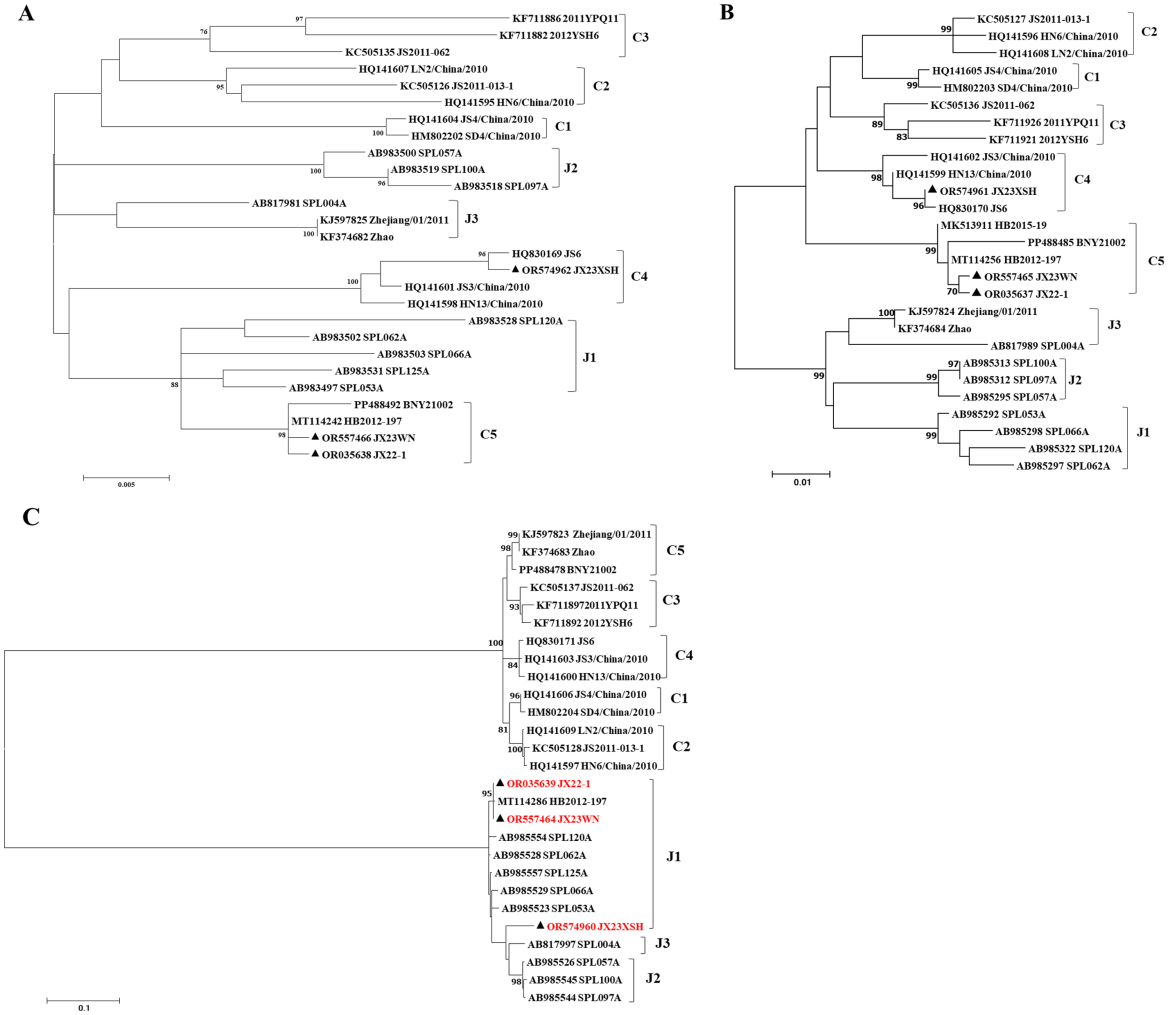
The maximum likelihood trees of the SFTSV genome in rectangular format for the partial L **(A)**, M **(B)**, and S **(C)** segments. The tree is drawn to scale, with branch lengths measured in the number of substitutions per site. Eight of the genotypes have been categorized, and the names of genotypes to the right side of trees (C1 to C5 and J1 to J3) are also shown. The values of the bootstrap percentage are shown next to the branches. The strains indicated by red-faced characters may have undergone segmental reassortment between genotypes. L, large; M, medium; S, small; SFTSV, severe fever with thrombocytopenia syndrome virus. The sequences with filled triangles are detected in this study.

Previous studies indicated that SFTSVs were clustered into the Japanese clade (including J1, J2, and J3 genotypes) and the Chinese clade (including C1, C2, C3, and C4 genotypes) (Shi et al., 2017; Yoshikawa et al., 2015). The phylogenetic analyses based on 878-bp L segment sequences revealed that sequences of the JX22-1 and JX23WN strains were clustered into an unidentified genotype (here assigned as C5) of the Chinese clade, and the JX23XSH strain into genotype C4 (Figure 2A). Phylogenetic analysis of 560-bp M segment sequences placed the JX22-1 and JX23WN strains on the genotype C5 branch, and the JX23XSH strain on the genotype C4 branch (Figure 2B). However, the phylogenetic analysis based on 577-bp S segment sequences revealed that all three SFTSV strains obtained in this study clustered with genotype J1 (Figure 2C), although none of the patients in Jiangxi had a history of travel to Japan prior to the onset of their disease. From the tree topology of the partial L, M, and S sequences, the three strains presented different evolutionary positions and changed among different clades. These results suggested that the S, M, and L segments of these strains may have derived from different ancestral genotypes, and they underwent segmental reassortment. The JX22-1 and JX23WN strains were subjected to segmental reassortment between genotype C5 and genotype J1, and the JX23XSH strain between the genotype C4 and the genotype J1 (Figure 2).

**Figure 3:**
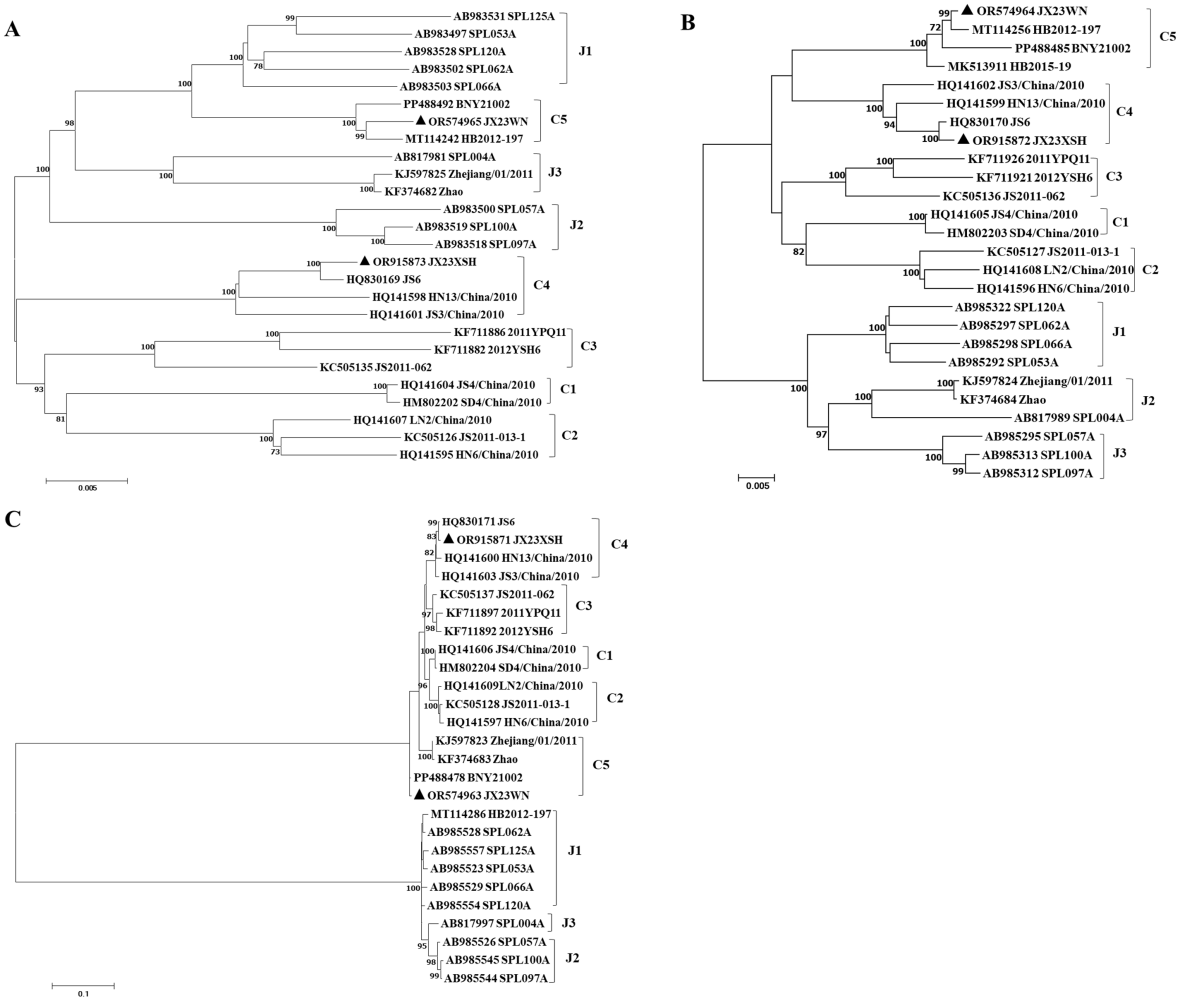
The maximum likelihood trees of the SFTSV genome in rectangular format for the complete L **(A)**, M **(B)**, and S **(C)** segments. The tree is drawn to scale, with branch lengths measured in the number of substitutions per site. Eight of the genotypes have been categorized, and the names of genotypes to the right side of trees (C1 to C5 and J1 to J3) are also shown. The values of the bootstrap percentage are shown next to the branches. L, large; M, medium; S, small; SFTSV, severe fever with thrombocytopenia syndrome virus. The sequences with filled triangles are detected in this study.

To avoid poor tree topology support with the partial S, M, and L segments, we further performed sequencing and analysis of the complete genome of SFTSV. The whole genome sequencing was successful in the JX23WN and JX23XSH strains, while the JX22-1 strain failed to be fully sequenced due to low SFTSV load. Identical to the tree topology of the partial L and M segments, the full-length L and M segment sequences of the JX23WN strain were also clustered into genotype C5, and the JX23XSH into genotype C4. However, different from the tree topology of the partial S segments, the full-length S segment sequences of the JX23WN and JX23XSH strains were grouped on the genotype C5 and C4 branches, respectively, not on the genotype J1 branch of the partial S tree (Figure 3). In combination with the L, M, and S phylogenetic tree of the partial and complete segments, these findings indicated that three strains in this study might have undergone segmental recombination between genotypes.

### 3.3 Recombination events have been detected on SFTSV in Jiangxi Province

**Figure 4:**
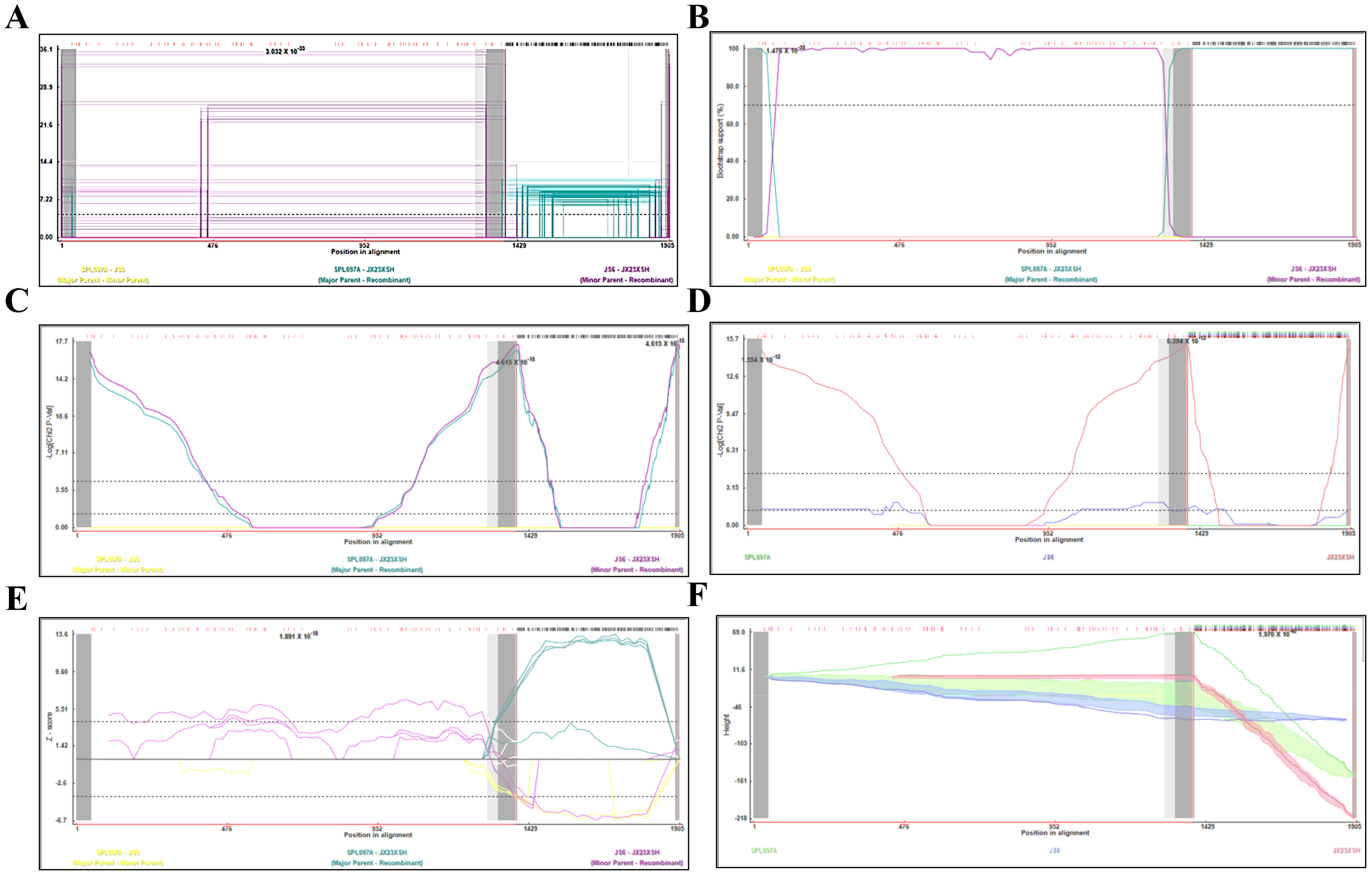
Plot of high scoring fragments for JX23XSH recombination signal based on the concatenation of the partial sequences of the L, M, and S segments. **(A)** GENECONV; **(B)** BootScan; **(C)** MaxChi; **(D)** Chimaera; **(E)** SiScan; **(F)** 3Seq. The left and right bounds of the red region indicate breakpoint positions suggested by the GENECONV method. Numbers in the panels are approximate *p*-values.

**Figure 5:**
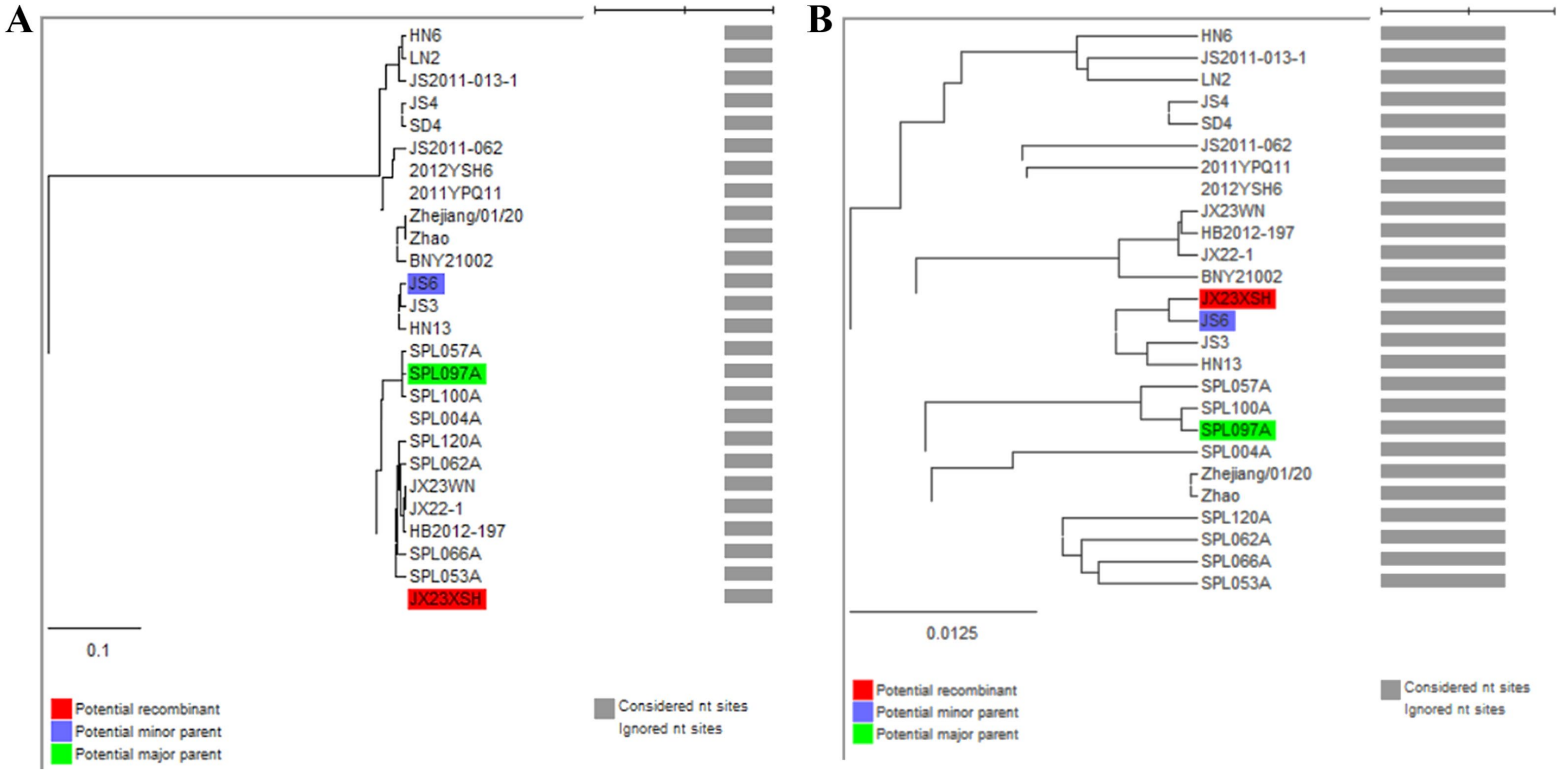
Unweighted pair-group method with arithmetic means (UPGMA) tree constructed with regions derived from major parent (1387–1897) **(A)** and minor parent (1–1,386 and 1,898–1,905) **(B)** of JX23XSH based on the concatenation of the partial sequences of the L, M, and S segments.

**Figure 6:**
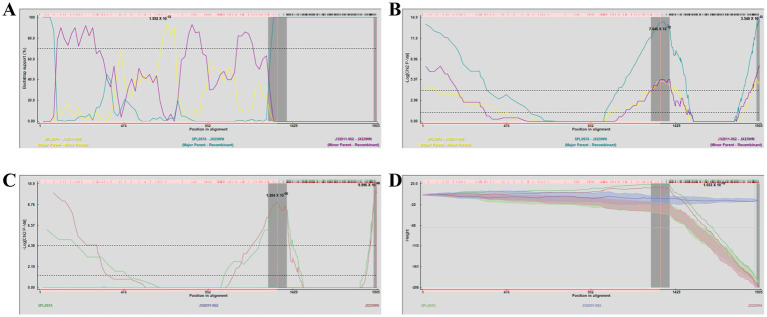
Plot of high scoring fragments for JX23WN recombination signal based on the concatenation of the partial sequences of the L, M, and S segments. **(A)** BootScan; **(B)** MaxChi; **(C)** Chimaera; **(D)** 3Seq. The left and right bounds of the red region indicate breakpoint positions suggested by the GENECONV method. Numbers in the panels are approximate *p*-values.

**Figure 7:**
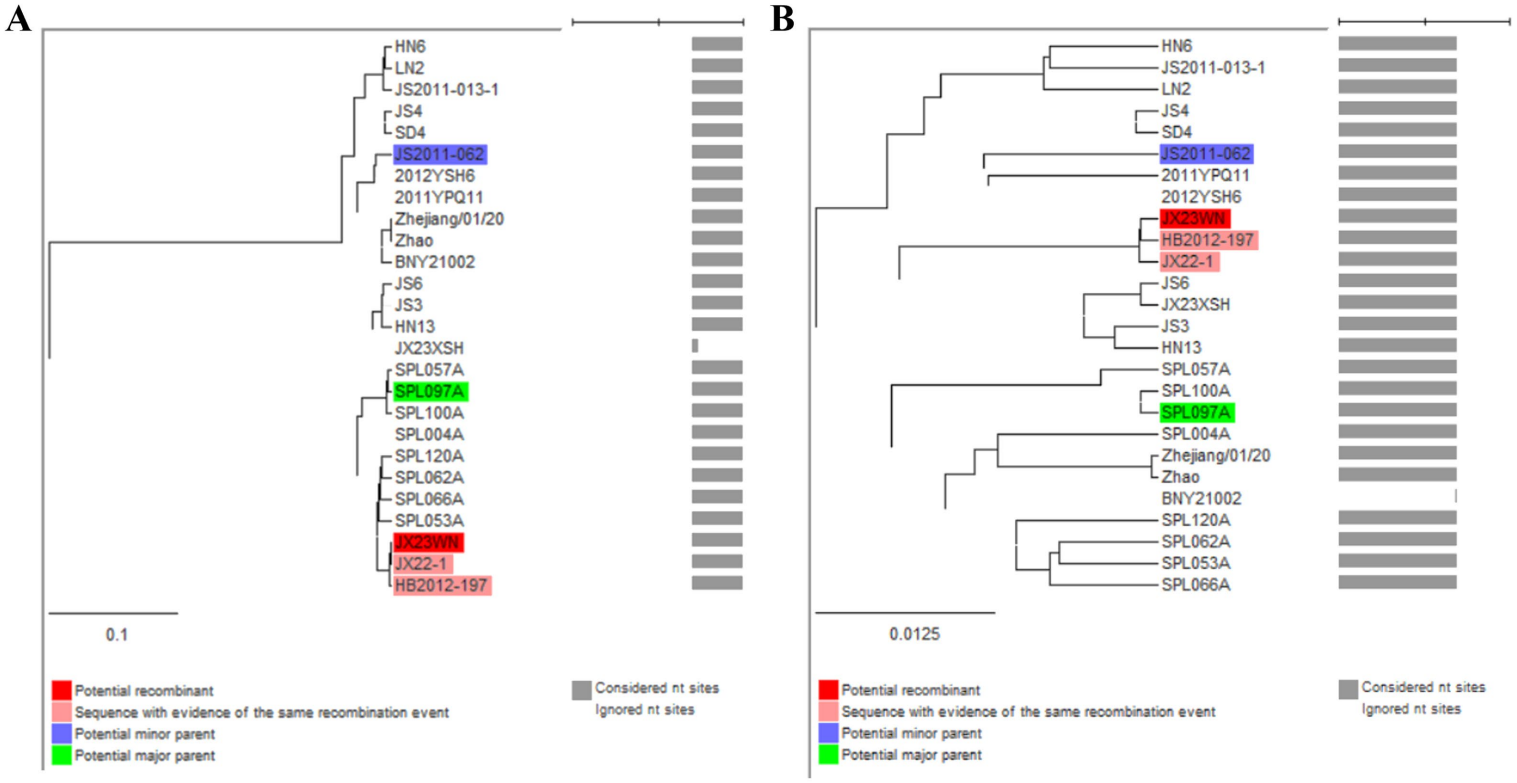
Unweighted pair-group method with arithmetic means tree constructed with regions derived from major parent (1,343–1,901) **(A)** and minor (1–1,342 and 1,902–1,905) **(B)** of JX23WN based on the concatenation of the partial sequences of the L, M, and S segments.

Based on the phylogenetic data, there were likely some recombination occurrences in SFTSVs from three patients residing in Jiangxi Province. To detect and further characterize recombination events, recombination signals and possible breakpoints were identified using six methods in the RDP package. The concatenated 878-bp L, 560-bp M, and 577-bp S sequences of SFTSV strains from Supplementary Dataset 1 were uploaded into the RDP package and run with default values. Consequently, two reassortment events appeared to occur between different clades (Table 3), and were found in the three strains with segments of inconsistent ancestral genotypes identified in the S, M, and L phylogenetic trees (Figure 2 and Table 3). The JX23XSH reassortment event was detected with high confidence (Table 3 and Figure 4). The event was detected by all the six methods with a significance level of *p*-value <0.05. RDPRCS was 0.71, which is greater than the threshold value 0.60 (Table 3 and Figure 4). The JX23XSH strain, from Xiushui County, Jiujiang City, Jiangxi Province, was designated as one reassortment event with the major parent (SPL097A, J2/J2/J2) and the minor parent (JS6, C4/C4/C4) (Table 3 and Figures 2, 4, 5). The GENECONV method identified the JX23XSH strain with two breakpoint positions at 1386 and 1897 nucleotide sites (Figures 4, 5). In comparison, the JX23WN reassortment event was detected with lower confidence. The event was detected by four methods with a significance level of *p*-value <0.05. RDPRCS was 0.50, falling within the range of 0.4 and 0.6 (Table 3 and Figure 6). Therefore, the event was considered a probable recombination. The JX22-1strain had the same recombination even as JX23WN strain. The JX23WN strain from Wuning County, Jiujiang City, Jiangxi Province, was designated as one reassortment event with the major parent (SPL097A, J2/J2/J2) and the minor parent (JS2011-062, C3/C3/C3) (Table 3 and Figures 2, 6, 7). The GENECONV method detected the strain with two breakpoint positions at 1,342 and 1,901 nucleotide sites (Figures 6, 7).

**Table 3:** SFTSV recombination events detected on partial length using the RDP package.

Recombination event	Major parent	Minor parent	RDPRCS	Tools	*p*-value
JX23XSH	SPL097A	JS6	0.714	GENECONV	3.03 × 10^−33^
				BootScan	1.48 × 10^−35^
				MaxChi	3.84 × 10^−15^
				Chimaera	5.12 × 10^−16^
				SiScan	5.25 × 10^−19^
				3Seq	1.97 × 10^−47^
JX23WN	JS2011-062	SPL097A	0.50	BootScan	4.52 × 10^−4^
				MaxChi	3.33 × 10^−12^
				Chimaera	1.16 × 10^−5^
				3Seq	2.47 × 10^−10^

**Figure 8:**
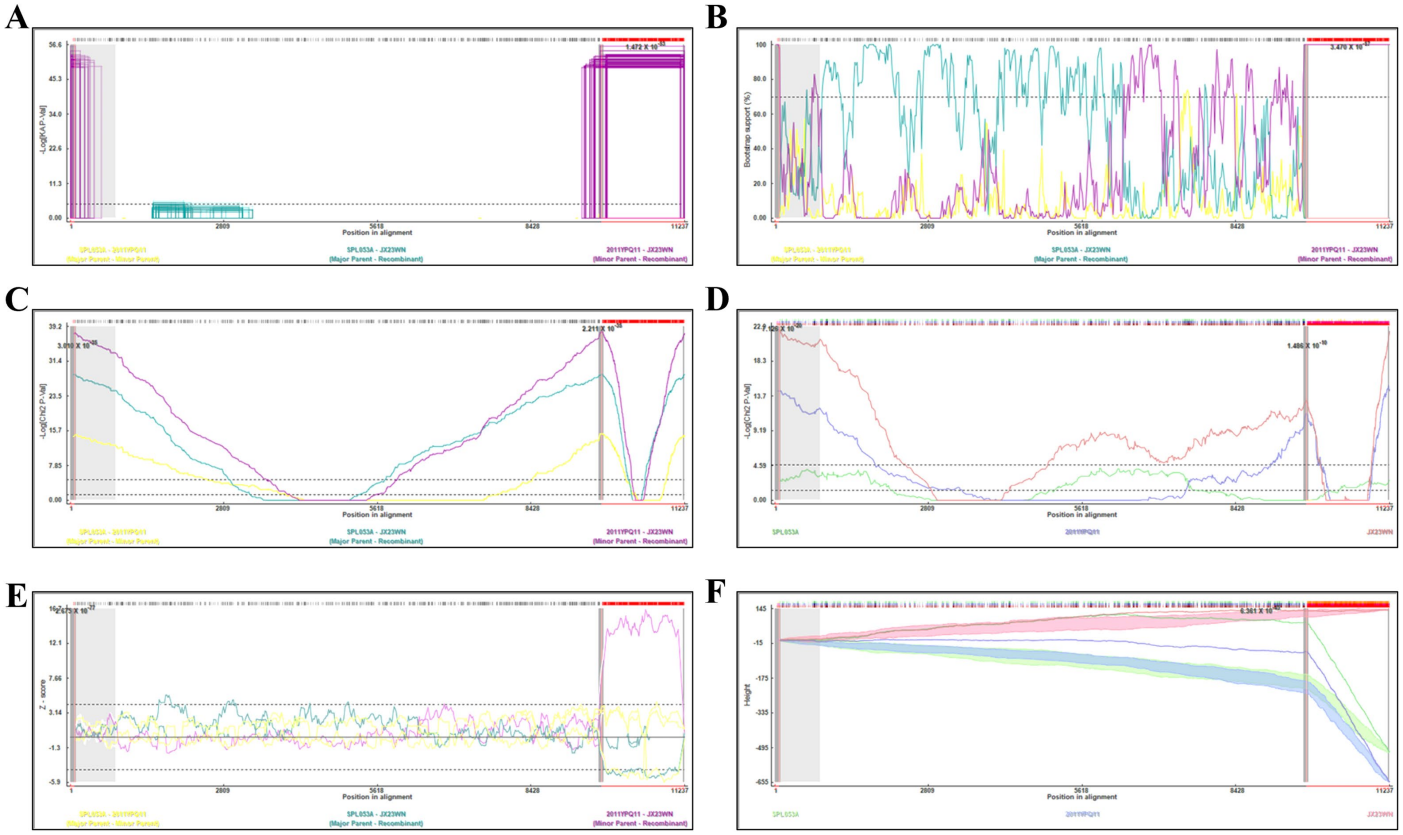
Plot of high scoring fragments for JX23WN recombination signal based on the concatenation of the complete sequences of the L, M, and S segments. **(A)**, GENECONV; **(B)** BootScan; **(C)** MaxChi; **(D)** Chimaera; **(E)** SiScan; **(F)** 3Seq. The left and right bounds of the red region indicate breakpoint positions suggested by the GENECONV method. Numbers in the panels are approximate *p*-values.

**Figure 9:**
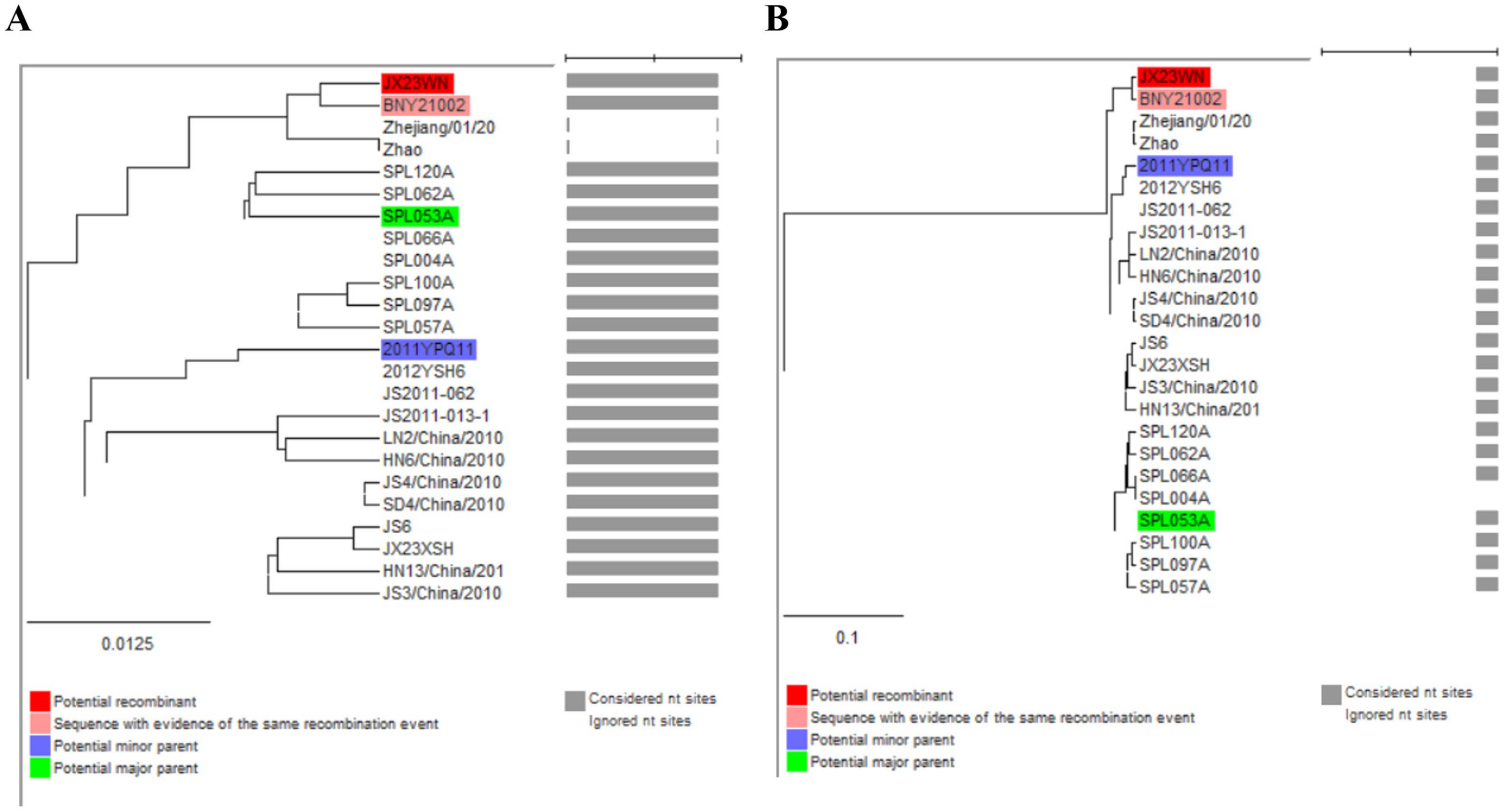
Unweighted pair-group method with arithmetic means tree constructed with regions derived from major parent (85–9714) **(A)** and minor (1–84 and 9,715–11,237) **(B)** of JX23WN based on the concatenation of the complete sequences of the L, M, and S segments.

Based on the concatenated full-length L, M, and S sequences of SFTSV strains from dataset 2, two predicted recombination events in SFTSV strains from Jiangxi province, including details of the recombinants and parents, are summarized in Table 4. The JX23WN recombination event was detected with moderate confidence (Table 4 and Figure 8). The event was detected by all the six methods with a significance level of *p*-value <0.05. However, RDPRCS was 0.41, falling within the range of 0.4 and 0.6 (Table 4 and Figure 8). Therefore, the event was considered a probable recombination. The JX23WN strain was designated as one probable recombination event with the minor parent (2011YPQ11, C3/C3/C3) and the major parent (SPL053A, J1/J1/J1) (Table 4 and Figures 8, 9). The recombination event contained parental sequences from different geographic areas, indicating the potential involvement of virus migration between different areas. The GENECONV method detected the strain with two breakpoint positions at 84 and 9,714 nucleotide sites (Figures 8, 9). The JX23XSH recombination event was detected with low confidence (Table 4). The event was detected by only two methods with a significance level of *p*-value <0.05 and RDPRCS was 0.42, falling within the range of 0.4 and 0.6 (Table 4). Therefore, the event was disregarded as a recombination.

**Table 4:** SFTSV recombination events detected on its full length using the RDP package.

Recombination event	Major parent	Minor parent	RDPRCS	Tools	*p*-value
JX23WN	SPL053A	2011YPQ11	0.41	GENECONV	8.16 × 10^−54^
				BootScan	2.64 × 10^−57^
				MaxChi	2.20 × 10^−10^
				Chimaera	3.54 × 10^−24^
				SiScan	8.29 × 10^−63^
				3Seq	6.36 × 10^−43^
JX23XSH	2012YSH6	SPL120A	0.42	GENECONV	1.89 × 10^−2^
				BootScan	1.83 × 10^−3^

## 4 Discussion and conclusion

The SFTS is a vector-borne disease transmitted primarily through the bite of infected ticks, particularly *H. longicornis* (Yu et al., 2011; Zhuang et al., 2018). The virus can also spread through direct contact with body fluids or blood from infected individuals (Tang et al., 2013). Symptoms of SFTS include the sudden onset of fever, gastrointestinal distress, bleeding, and low platelet count, which can lead to life-threatening complications if not promptly treated (Liu et al., 2014; Yu et al., 2011). Currently, the epidemic status of SFTSV in China is characterized by widespread presence across various provinces, with significant numbers of cases reported in region such as Henan, Hubei, Jiangsu, Zhejiang, Anhui, and Shandong (Li et al., 2022). Despite bordering with the epidemic Hubei, Anhui, and Zhejiang, Jiangxi has only a few cases, ranging from zero to two patients during 2011 and 2018 (Qian, 2020). However, in recent years patients with SFTSV infection have been increasingly found in the Mufu mountains of Jiujiang, Jiangxi. Here, we found four SFTS patients in Jiujiang, Jiangxi though we failed to sequence the viral genome from the fourth patient. We inferred the failure from low virus load. The first, second and third patients were detected positive for SFTSV with *C*t value of 32, 26, and 30, respectively. They had at least 1,000-fold higher virus loads than that of the fourth patient, whose serum was positive in qRT-PCRs for SFTSV with *C*t value of 35 (unpublished data).

The PCR tests for SFTSV infection in high-risk humans, animals and ticks surrounding these patients yielded negative results. However, two out of the 56 serum samples tested strongly positive for immunoglobulin G (IgG) antibody against SFTSV using ELISA, with one of samples belonging to the wife of patient 2. In addition, a serum sample from one of the 12 dogs tested was also positive for anti-SFTSV IgG (unpublished data). Our results indicate that Jiujiang may have natural conditions for SFTSV circulation.

The SFTSV strains have highly conservative L segment (95.81–99.64%), moderately conservative M segment (93.66–99.70%), and yet variable S segment (26.95–99.48%). Furthermore, this virus also exchanges genes between different strains. Therefore, SFTSV is reported as a rapidly evolutionary virus, and it currently has at least 10 genotypes at present. These genotypes consist of genotype C1–C4, primarily from China, and J1–J3, mainly from Japan (Ding and He, 2012; Lv et al., 2017; Wu et al., 2021). The evolutionary forces shaping these viruses are complex and multifaceted. SFTSV has high mutation rates (~10–4 substitutions/site/year) during its replication due to the lack of proofreading capabilities in their RNA-dependent RNA polymerases (RdRps) (Lam et al., 2013). Although recombination is uncommon in SFTSV, it can occur through the co-infection of a host cell by two or more genetically distinct viruses. Recombination can result in the exchange of genetic information and scientists have reported a recombination event between two strains JF837594/JS2007-01 and HM802203/SD4/China/2010, at the 503 and 1,410 nucleotide sites of M segment, leading to the generation of a novel genotype HQ642767/BX-2010/Henan/CHN with unique combinations of traits (Ding and He, 2012). SFTSV is one of segmented-genome viruses, with its genome divided into three segments. Reassortment can occur when two or more genetically distinct virus strains infect the same host cell simultaneously. During this process, viral segments from different strains can be shuffled and reassorted, leading to the generation of novel virus genotypes. For instance, the strain YSC3 has L and M segments from genotype C2, and exchanges its S segment from C2 to C4 (Lv et al., 2017). There are two reassortment patterns in SFTSV: single reassortment and multiple reassortment. The single reassortment occurs when a strain has one segment belonging to one genotype and the other two segments belonging to a distinct genotype. Wu et al. (2021) found single reassortment in 13 strains, with 6 reassortment events involving the S segment, 3 events involving the M segment, and 4 events involving the L segment. Multiple reassortment occurs when all the three segments belong to a different genotype. The strains AHL, NB-32, KADGH4, and ZJ2014-01 were involved in multiple reassortment events (Wu et al., 2021). Although some cases had been found in Jiangxi, there are only six strains of SFTSV genomes from Jiangxi Province searched in National Center for Biotechnology information (NCBI). These strains include three strains analyzed in this study. Up to now no evolutionary characteristics have been exposed on the virus from Jiangxi. Here, based on the phylogenetic analysis of the complete L, M, and S segments, two SFTSV genotypes were detected in Jiujiang city, Jiangxi Province: C4 and C5 genotypes, respectively. Further recombination analysis, as well as phylogenetic tree of the partial S segment, suggested that the strain JX23WN underwent genetic recombination. It evolved from the strain 2011YPQ11 as the minor parent and the strain SPL053A as the major parent. However, coinfection experiments need to be conducted to confirm whether recombination can occur between the strain 2011YPQ11 and the strain SPL053A to generate the strain JX23WN.

Although we sampled various specimens related to SFTSV infection, including patients, high risk humans, ticks and animals, and conducted a phylogenetic study to investigate the molecular characteristics of SFTSVs, we continue to recognize the significance of expanding our sample collection and acquiring additional SFTSV strains. Ticks collected by flagging vegetation and from domestic animals were negative for SFTSV sequences in the present study, and this might be attributed to the limited number of vector tick samples that were collected. Conducting surveying SFTSV infection in ticks, animals, and high-risk individuals only after case reporting could have potentially introduced bias into the determination of SFTSV distribution. Moreover, all of the ticks were extracted from domestic animals (goats, dogs, and cats), raising the likelihood of excluding ticks associated with wild animals within the surveyed locations. The SFTS virus is primarily transmitted by certain species of ticks, such as *H. longicornis* which is the dominant tick species in the survey areas (Zhuang et al., 2018). However, the prevalence of the virus in tick species can vary depending on various factors, including geographic location and seasonal variations. For example, the SFTSV was detected in 5.4% of *H. longicornis* specimens from Shandong, Henan and Hubei provinces in China (Yu et al., 2011). However, only 0.5% of tested specimens from Korea showed the presence of the SFTSV (Park et al., 2014). To accurately assess the presence of the SFTS virus in ticks, in the future we will collect an adequate number of samples including wild animals, tick vectors, and domestic animals from different locations and time periods in the Mufu mountains. This will ensure a representative sample size that can provide reliable data on the virus’s prevalence and molecular characteristics. In recent years, there have been many annual SFTS cases in the Mufu mountains of Jiangxi, and the morbidity is increasing. More effort should be made to systematically survey the epidemic of SFTS, its vectors and hosts, to elucidate the mechanism of the natural focal disease.

Although our study evaluated the recombination signals of SFTSVs in the Mufu mountains of Jiangxi, we could additionally obtain genetic sequences from Jiujiang and other areas of Jiangxi Province to better understand the evolutionary profiles in this province. A comprehensive understanding of evolutionary force and pathway will provide significant benefits in combating local SFTSV pandemics.

Taken together, SFTSV presence was found in Jiangxi. Furthermore, the sequence results demonstrate that C4 and C5 genotypes are present, and genotype C5 undergoes genetic recombination in Jiangxi Province of Central China.

## Data Availability

The datasets presented in this study can be found in online repositories. The names of the repository/repositories and accession number(s) can be found in the article/Supplementary material.

## References

[ref1] Casel M. A. Park S. J. Choi Y. K. (2021). Severe fever with thrombocytopenia syndrome virus: emerging novel phlebovirus and their control strategy. Exp. Mol. Med. 53, 713–722. doi: 10.1038/s12276-021-00610-1, PMID: 33953322 PMC8178303

[ref2] Chen Z. Yang X. J. (2021). Tick systematics. Beijing: Science Press.

[ref3] Ding N. Z. He C. Q. (2012). Discovery of severe fever with thrombocytopenia syndrome bunyavirus strains originating from intragenic recombination. J. Virol. 86, 12426–12430. doi: 10.1128/JVI.01317-12, PMID: 22933273 PMC3486477

[ref4] Hu B. Cai K. Liu M. Li W. Xu J. Qiu F.. (2018). Laboratory detection and molecular phylogenetic analysis of severe fever with thrombocytopenia syndrome virus in Hubei Province, Central China. Arch. Virol. 163, 3243–3254. doi: 10.1007/s00705-018-3993-5, PMID: 30136250

[ref5] Kimura M. (1980). A simple method for estimating evolutionary rates of base substitutions through comparative studies of nucleotide sequences. J. Mol. Evol. 16, 111–120. doi: 10.1007/BF01731581, PMID: 7463489

[ref6] Lam T. T. Y. Liu W. Bowden T. A. Cui N. Zhuang L. Liu K.. (2013). Evolutionary and molecular analysis of the emergent severe fever with thrombocytopenia syndrome virus. Epidemics 5, 1–10. doi: 10.1016/j.epidem.2012.09.002, PMID: 23438426 PMC4330987

[ref7] Li J. C. Zhao J. Li H. Fang L. Q. Liu W. (2022). Epidemiology, clinical characteristics, and treatment of severe fever with thrombocytopenia syndrome. Inf. Med. 1, 40–49. doi: 10.1016/j.imj.2021.10.001, PMID: 38074982 PMC10699716

[ref8] Liu Q. He B. Huang S. Y. Wei F. Zhu X. Q. (2014). Severe fever with thrombocytopenia syndrome, an emerging tick-borne zoonosis. Lancet Infect. Dis. 14, 763–772. doi: 10.1016/S1473-3099(14)70718-2 24837566

[ref9] Lv J. Wu S. Zhang Y. Chen Y. Feng C. Yuan X.. (2014). Assessment of four DNA fragments (COI, 16S rDNA, ITS2, 12S rDNA) for species identification of the Ixodida (Acari: Ixodida). Parasit. Vectors 7:93. doi: 10.1186/1756-3305-7-93, PMID: 24589289 PMC3945964

[ref10] Lv J. Zhang H. Tian L. Zhang R. L. Zhang Z. J. Li J.. (2017). Novel sub-lineages, recombinants and reassortants of severe fever with thrombocytopenia syndrome virus. Ticks Tick Borne Dis 8, 385–390. doi: 10.1016/j.ttbdis.2016.12.015, PMID: 28117273

[ref11] Martin D. P. Murrell B. Golden M. Khoosal A. Muhire B. (2015). RDP4: detection and analysis of recombination patterns in virus genomes. Virus Ecol 1:vev003. doi: 10.1093/ve/vev003, PMID: 27774277 PMC5014473

[ref12] Miao D. Liu M. J. Wang Y. X. Ren X. Lu Q. B. Zhao G. P.. (2021). Epidemiology and ecology of severe fever with thrombocytopenia syndrome in China, 2010–2018. Clin. Infect. Dis. 73, e3851–e3858. doi: 10.1093/cid/ciaa1561, PMID: 33068430 PMC8664468

[ref13] Nagarajan N. Kingsford C. (2011). GiRaF: robust, computational identification of influenza reassortments via graph mining. Nucleic Acids Res. 39:e34. doi: 10.1093/nar/gkq1232, PMID: 21177643 PMC3064795

[ref14] Park S. W. Song B. G. Shin E. H. Yun S. M. Han M. G. Park M. Y.. (2014). Prevalence of severe fever with thrombocytopenia syndrome virus in *Haemaphysalis longicornis* ticks in South Korea. Ticks Tick Borne Dis 5, 975–977. doi: 10.1016/j.ttbdis.2014.07.020 25164614

[ref15] Qian J. (2020). Studies on significant epidemiological characteristics of severe fever with thrombocytopenia syndrome and coronavirus disease 2019 in mainland China. Beijing: Peking Union Medical College.

[ref16] Saitou N. Nei M. (1987). The neighbor-joining method: a new method for reconstructing phylogenetic trees. Mol. Biol. Evol. 4, 406–425. doi: 10.1093/oxfordjournals.molbev.a040454, PMID: 3447015

[ref17] Shi J. Hu S. Liu X. Yang J. Liu D. Wu L.. (2017). Migration, recombination, and reassortment are involved in the evolution of severe fever with thrombocytopenia syndrome bunyavirus. Infect. Genet. Evol. 47, 109–117. doi: 10.1016/j.meegid.2016.11.015 27884653

[ref18] Tamura K. Stecher G. Peterson D. Filipski A. Kumar S. (2013). MEGA6: molecular evolutionary genetics analysis version 6.0. Mol. Biol. Evol. 30, 2725–2729. doi: 10.1093/molbev/mst197 24132122 PMC3840312

[ref19] Tang X. Y. Wu W. L. Wang H. F. Du Y. H. Liu L. C. Kang K.. (2013). Human-to-human transmission of severe fever with thrombocytopenia syndrome Bunyavirus through contact with infectious blood. J. Infect. Dis. 207, 736–739. doi: 10.1093/infdis/jis748 23225899

[ref20] Wu X. L. Li M. Y. Zhang Y. F. Liang B. Y. Shi J. M. Fang Y. H.. (2021). Novel SFTSV phylogeny reveals new reassortment events and migration routes. Virol. Sin. 36, 300–310. doi: 10.1007/s12250-020-00289-0, PMID: 32960400 PMC8087752

[ref21] Yoshikawa T. Shimojima M. Fukushi S. Tani H. Fukuma A. Taniguchi S.. (2015). Phylogenetic and geographic relationships of severe fever with thrombocytopenia syndrome virus in China, South Korea, and Japan. J. Infect. Dis. 212, 889–898. doi: 10.1093/infdis/jiv144 25762790

[ref22] Yu X. J. Liang M. F. Zhang S. Y. Liu Y. Li J. D. Sun Y. L.. (2011). Fever with thrombocytopenia associated with a novel bunyavirus in China. N. Engl. J. Med. 364, 1523–1532. doi: 10.1056/NEJMoa1010095, PMID: 21410387 PMC3113718

[ref23] Zhuang L. Sun Y. Cui X. M. Tang F. Hu J. G. Wang L. Y.. (2018). Transmission of severe fever with thrombocytopenia syndrome virus by *Haemaphysalis longicornis* ticks, China. Emerg. Infect. Dis. 24, 868–871. doi: 10.3201/eid2405.151435, PMID: 29664718 PMC5938789

