# Supplementary material for: Severe fever with thrombocytopenia syndrome virus was found in Northern Jiangxi Province, China
Source: Front Microbiol. 2024 Dec 4;15:1500146. doi: 10.3389/fmicb.2024.1500146 (PMC11666098; doi:10.3389/fmicb.2024.1500146)
Supplement: Supplementary file 1 [file Data_Sheet_1.ZIP › Supplementary materials/Supplementary Table 1.docx]

Supplementary Table 1. Epidemiological characteristics, clinical symptoms and laboratory findings of the four patients with severe fever with thrombocytopenia syndrome on admission.

| **Basic information** | Patient 1 | Patient 2 | Patient 3 | Patient 4 |
| --- | --- | --- | --- | --- |
| Sex | Female | Male | Female | Male |
| Age (years) | 48 | 68 | 55 | 49 |
| Occupation | Frog raiser, tea picker | Maize planter^⁎^ | Bamboo shoot digger, tea picker | Fresh water fish hunter |
| Hospitalization — days | 23 | 64 | 23 | 16 |
| Chronic diseases | Hepatitis B  (40 years) | Hypertension | Gastropathy | Bronchitis |
| Death or survival after discharge | Survival | Survival | Survival | Survival |
| **Epidemiological characteristics** | | | | |
| Tick bites | Yes (high frequency) | Yes | No | Yes |
| Days between known tick bite and illness onset | About 40 | About 20 | NA | About 10 |
| Tick presence in surroundings | Yes (common) | Yes | Yes | Yes |
| Other arthropod bites | Mosquito, flea | NA | Mosquito | Mosquito, flea |
| Animal raising | Four dogs, one cat | No | Rodent infestation | Dog |
| Animal contact | Yes | No | Chicken and duck | Yes |
| Animals with ticks | Yes | No | No | Yes |
| **Clinical symptoms** | | | | |
| Fever | Yes (39.9℃) | Yes  (38.0℃) | Yes | Yes  (39℃) |
| Anorexia | No | Yes | Yes | Yes |
| Fatigue | No | Yes | Yes | Yes |
| Nausea | No | Yes | Yes | Yes |
| Abdominal pain or tenderness | No | Yes | Yes | Yes |
| Vomiting | Yes | Yes | Yes | Yes |
| Malaise | Yes | Yes | Yes | Yes |
| Diarrhea | Yes | Yes | Yes | Yes |
| Lymphadenopathy | Yes | No | No | No |
| Myalgia | No | Yes | No | Yes |
| Headache | No | Yes | No | Yes |
| Throat congestion | No | No | No | No |
| Cough | No | No | Yes | Yes |
| Conjunctival congestion | No | No | No | No |
| Petechiae | No | No | Yes | No |
| Apathy | No | Yes | Yes | Yes |
| Slurred speech | No | Yes | No | No |
| Coma | No | Yes | No | No |
| **Hematologic test** | | | | |
| Leukocyte count — ×10^9^ /liter | 0.75↓ | 1.59↓ | 1.91↓ | 2.90↓ |
| Lymphocyte count —×10^9^ /liter | 0. 12↓ | 0.37↓ | 1.05↓ | 0.66↓ |
| Neutrophil count —×10^9^ /liter | 0. 62↓ | 1.16↓ | 0.71↓ | 2.17 |
| Hemoglobin —g/liter | 102↓ | 143 | 123 | 171↑ |
| Platelet count —×10^9^ /liter | 19↓ | 35↓ | 25↓ | 68↓ |
| **Biochemical test** | | | | |
| Alanine aminotransferase — U/liter | 176.7↑ | 300.1↑ | 196.4↑ | 63.3↑ |
| Aspartate aminotransferase— U/liter | 445.5↑ | 821.5↑ | 815.3↑ | 153.7↑ |
| Activated partial thromboplastin time— sec | NA | NA | 67.2↑ | 46.1↑ |
| Thrombin time— sec | NA | NA | 122.4↑ | NA |
| Creatine kinase — U/liter | NA | NA | NA | 599.4↑ |
| Creatine kinase-MB— U/liter | NA | NA | NA | 56.6↑ |
| **Urinalysis** | | | | |
| Proteinuria | No | No | No | NA |
| Hematuria | No | + | No | NA |
| **Fecal analysis** | | | | |
| Fecal occult blood | No | No | + | NA |

⁎, he also frequently mows grass to feed fish.

Patient 1 has a dead dog with loose feces.

NA, not available.
